# *QuickStats:* Percentage* of Adults Aged ≥18 Years Who Sleep <7 Hours on Average in a 24-Hour Period,^†^ by Sex and Age Group — National Health Interview Survey,^§^ United States, 2020

**DOI:** 10.15585/mmwr.mm7110a6

**Published:** 2022-03-11

**Figure:**
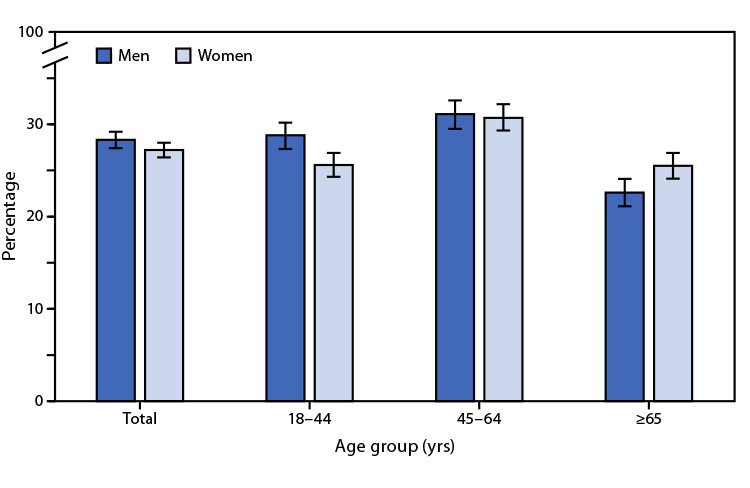
Overall, 28.3% of men and 27.2% of women aged ≥18 years slept <7 hours on average within a 24-hour period. Among persons aged 18–44 years, men (28.8%) were more likely to sleep <7 hours compared with women (25.6%). Among adults aged 45–64 years, the percentage was similar for men (31.1%) and women (30.7%). However, among those aged ≥65 years, women (25.5%) were more likely than men (22.6%) to sleep <7 hours.

**Source:** National Center for Health Statistics, National Health Interview Survey, 2020. https://www.cdc.gov/nchs/nhis/index.htm

